# Urban versus rural differences in the occurrence of hip fractures in Japan’s Kyoto prefecture during 2008–2010: a comparison of femoral neck and trochanteric fractures

**DOI:** 10.1186/1471-2474-14-304

**Published:** 2013-10-25

**Authors:** Motoyuki Horii, Hiroyoshi Fujiwara, Takumi Ikeda, Keiichiro Ueshima, Kazuya Ikoma, Toshiharu Shirai, Ryu Terauchi, Masateru Nagae, Nagato Kuriyama, Toshikazu Kubo

**Affiliations:** 1 Department of Orthopaedics, Graduate School of Medical Science, Kyoto Prefectural University of Medicine, Kyoto, Japan; 2 Department of Epidemiology for Community Health and Medicine, Graduate School of Medical Science, Kyoto Prefectural University of Medicine, Kyoto, Japan

**Keywords:** Hip fracture, Femoral neck fracture, Trochanteric fracture, Urban, Rural

## Abstract

**Background:**

To investigate the differences in the characteristics of femoral neck and trochanteric fractures between urban and rural areas of Kyoto Prefecture in Japan.

**Methods:**

Fracture type (neck vs. trochanteric), age, sex, place where fracture occurred (indoors vs. outdoors), and cause of injury were surveyed among patients aged ≥65 years who sustained hip fractures between 2008 and 2010 and who were treated at 1 of 13 participating hospitals (5 urban, 8 rural). The ratio of sick beds to total number of beds at the participating hospitals was 19.6% (2,188/11,158) in the urban area and 34.9% (1,963/5,623) in the rural area. We also investigated the incidence of hip fracture in Tango medical district as a representative rural area.

**Results:**

There were 1,346 neck (mean age, 82.4 years) and 1,606 trochanteric fractures (mean age, 85.0 years). The ratio of neck to trochanteric fractures was higher in the urban area than in the rural area in all age groups (65–74, 75–84, and ≥ 85 years). There were no apparent differences in place or cause of injury. The incidence of hip fracture in the women of Tango medical district was lower than the national average.

**Conclusions:**

There was a difference in the ratio of neck to trochanteric fractures between urban and rural areas. This difference is estimated to be caused by the high and low incidence of neck fracture in urban and rural areas, respectively.

## Background

In many epidemiologic surveys, femoral neck fractures (hereafter referred to as neck fractures) and trochanteric fractures are treated indiscriminately. However, there are differences in their international [1,2] and age-related [3-8] incidences. For example, Northern Europe and Africa [5,8,9] have a higher incidence of neck fractures, while Japan [10-12] and the Mediterranean region [13,14] have a higher incidence of trochanteric fractures.

Kyoto Prefecture, located in Midwestern Japan, is long (120 km) in the north-and-south direction and is divided into 6 medical districts [15]. One of these districts, Kyoto Otokuni, is an urban area that includes Kyoto city and comprises approximately 18.9% (871 km^2^) of the area of Kyoto Prefecture and approximately 61.7% of the population (1,630,000). There are considerable differences in the living environments between the urban and rural areas (Table 1). Based on our investigation of Kyoto Prefecture in 2008, it was suspected that there may be a difference in the ratio of neck to trochanteric fractures (N/T ratio) between the urban and rural areas [15]. However, which type of fracture has a higher or lower incidence in which area is unclear. Generally, determination of fracture incidence in urban areas is difficult because many patients are not confined to that region for treatment. In rural areas, however, it is easier to estimate the ratio because patients tend to receive treatment at limited medical institutions within the region of their residence. The incidence ratio of hip fractures within Kyoto Prefecture is clear from a previous national survey [16]. Therefore, if incidence in the rural area is clarified, that of the urban area can be estimated.

**Table 1:** Prevalence of neck fracture, mean ages, and indices of regional environment and background in each district

**Area**		**Urban**	**Rural**				
**Medical district**			As a whole^*^				
		**Kyoto-Otokuni**		**Tango**	**Chutan**	**Nantan**	**Yamashiro Kita**
Neck fracture,%^a^		51.7	41.9	34.2	42.5	46.2	41.6
Mean age, y	Hip fracture^b^	83.1	84.2	85.3	84.2	83.5	84.3
	Neck fracture^c^	81.7	82.9	83.2	82.8	82.6	83.3
	Trochanteric fracture^d^	84.6	85.2	86.4	85.2	84.3	85.0
Age distribution,%^†^	65-74 y	53.2	51.8	43.9	45.6	49.5	59.0
	75-84 y	34.2	34.7	38.8	38.5	36.0	30.6
	≥ 85 y	12.6	13.5	17.3	15.9	14.5	10.4
Elderly (≥65 years old) living alone,%^†^		20.9	14.5	14.2	16.6	12.3	14.2
Elderly (≥65 years old) living with aged spouse only,%^†^		44.4	46.9	52.7	49.1	46.0	44.6
Population density,/km^2†^		1,886.0	124.8	164.4	125.3	1,729.9	434.9
Primary industry’s working population among persons at work,%	Total (1985)^‡^	1.4	9.9	14.8	15.0	14.1	3.2
	Total (2010)^†^	0.8	4.3	8.1	5.7	6.8	1.8
Primary industry’s working population,%	≥ 65 y (2010)^†^	0.7	4.9	7.2	7.1	8.0	1.7
	≥ 75 y (2010)^†^	0.6	4.8	6.2	6.6	7.0	1.7
Doctors/100,000 people^§^	Total	359.2	174.3	152.6	209.2	170.2	164.9
	Orthopedics	22.4	15.4	17.2	15.2	17.4	14.4

^a^Statistical differences were seen between urban and rural areas as a whole (*P* < .0001), Tango and Chutan (*P* < .05), Tango and Nantan (*P* < .005), Tango and Kyoto-Otokuni (*P* < .001), Chutan and Kyoto-Otokuni (*P* < .001), and Kyoto-Otokuni and Yamashiro Kita (*P* < .001).

^b^Statistical differences were seen between urban and rural areas as a whole (*P* < .0005), Tango and Chutan (*P* < .05), Tango and Nantan (*P* < .005), Tango and Kyoto-Otokuni (*P* < .0001), Chutan and Kyoto-Otokuni (*P* < .005), and Kyoto-Otokuni and Yamashiro Kita (*P* < .01).

^c^Statistical differences were seen between Chutan and Kyoto-Otokuni (*P* < .05) and Kyoto-Otokuni and Yamashiro Kita (*P* < .05). No statistical difference was seen between urban and rural areas.

^d^Statistical differences were seen between Tango and Nantan (*P* < .005), Tango and Kyoto-Otokuni (*P* < .005), and Tango and Yamashiro Kita (*P* < .05). No statistical difference was seen between urban and rural areas.

*Yamashiro Minami medical district was excluded.

^†^Source: National Census, 2010.

^‡^Source: National Census, 1985.

^§^Source: Doctors, Dentists and Pharmacists Survey, 2010.

We retrospectively surveyed the difference in N/T ratio between rural and urban areas in Kyoto Prefecture over 3 years. We also investigated incidence of hip fracture in one representative rural medical district.

## Methods

This was a retrospective, multicenter, observational study. The institutions conducting the investigations were 13 hospitals from 5 of 6 medical districts in Kyoto Prefecture. All are Japanese Orthopaedic Association (JOA)–authorized hospitals. Five of the 13 hospitals are located in the Kyoto Otokuni district, an urban area, while the other 8 are located in a rural area. University hospitals (Kyoto Prefectural University and Kyoto University) were excluded because the suspected number of hip fractures treated at these hospitals was small (approximately 10 cases/year) considering the number of beds (1,065 and 1,182, respectively). The ratio of sick beds for acute-term care to total number of beds at the investigated hospitals was 19.6% (2,188/11,158) in the urban area and 34.9% (1,963/5,623) in the rural area.

The subjects of the study were patients aged ≥65 years who sustained hip fractures between January 1, 2008, and December 31, 2010, and who were treated at one of the participating hospitals. Patients with isolated fractures of the greater trochanter, subtrochanteric fracture, or pathologic fracture were excluded. Registration forms according to the nationwide survey of the JOA [10] were sent to the 13 institutions by mail, and registration was performed by doctors at each hospital according to their hospital records. Variables examined included sex, age, affected side, fracture type (neck vs. trochanteric), place where the fracture occurred (indoors vs. outdoors), and cause of injury. Cause of injury was divided into 6 categories according to the nationwide survey: in bed, simple fall, fall on stairs, traffic accident, could not be recalled, and unknown [10].

Age, place where fracture occurred, and cause of injury were compared between neck and trochanteric fractures. Differences in place and cause were also compared between the urban and rural areas within 3 age groups: 65–74, 75–84, and ≥85 years.

We also investigated the hip fracture incidence in Tango medical district, part of the rural area that was investigated within Kyoto Prefecture. Kyoto Prefectural Yosanoumi Hospital—a participating hospital—is the only institution that covered 1 city and 2 towns in Tango medical district, which has a population of 45,812 and an area of 338 km^2^. Because no other hospitals treat orthopedic injuries within the region, most patients with hip fracture in this area receive treatment at this hospital. Patients in adjacent areas are treated at other hospitals in each district. For comparison with the nationwide survey [16], incidence of hip fractures in each sex was calculated within the following age groups: 70–79, 80–89, and ≥90 years. In addition, age-related incidence of neck and trochanteric fractures was compared within each sex.

Ethical approval was obtained from the ethics committee of Kyoto Prefectural University of Medicine.

Chi-square test was used to compare differences in N/T ratio among the medical districts. For age comparison, Student’s *t* test was used. A P value of < 0.05 was regarded as significant. Statistical analyses were conducted with StatFlex Ver. 6.0 (Artech Co., Ltd., Osaka, Japan).

## Results

The survey results indicated a total of 2,952 hip fractures (2,388 women, 80.1%) during the 3 years: 1,006 cases in 2008, 961 in 2009, and 985 in 2010. The mean age (±SD) was 83.8 (±7.3) years. With regard to fracture type, 1,606 cases (56.0%) of trochanteric fractures and 1,346 cases (44.0%) of neck fracture were reported. The mean patient age (±SD) for each fracture type was 85.0 (±7.0) and 82.4 (±7.4) years, respectively. The mean patient age for trochanteric fractures was significantly higher than that for neck fractures (P = 0.0000). The ratio of women with trochanteric and neck fractures was 79.5% (1,277 cases) and 82.5% (1,111 cases), respectively. The ratio of women was significantly higher than that of men for both fracture types, compared with the ≥65 years male-to-female population ratio (247,262:336,301; National Census, 2010) in the subject area (P = 0.0000). The right side was the affected side for hip fractures in 48.6% of patients, for neck fractures in 49.7%, and for trochanteric fractures in 47.8%.

Table 1 shows the ages and incident numbers according to fracture type in each medical district. The urban area had a higher incidence of neck fractures (51.7%) than trochanteric fractures, while the rural area had a higher incidence of trochanteric fractures (58.2%) than neck fractures. There was a significant difference in the N/T ratio between the urban and rural areas (P = 0.0000). The ratios of trochanteric fracture in 2008, 2009, and 2010 were 47.1%, 48.2%, and 49.6% in the urban area and 58.3%, 56.0%, and 60.1% in the rural area, respectively.

The incident numbers of neck and trochanteric fractures within each age group were 209 and 137 for 65–74 years of age, 589 and 577 for 75–84 years of age, and 548 and 892 for ≥ 85 years of age, respectively (Table 2).

**Table 2:** Differences in place and cause of fracture between neck and trochanteric fractures in each age group

**Age group, y**		**65–74**		**74–84**		≥**85**	
		**Neck fracture**	**Trochanteric fracture**	**Neck fracture**	**Trochanteric fracture**	**Neck fracture**	**Trochanteric fracture**
No. of hip fractures		209	137	589	577	548	892
Neck-to-trochanteric fracture ratio		1.53		1.02		0.61	
Place of injury, n (%)							
	Indoors	113 (54.1)	78 (56.9)	380 (64.5)	405 (70.2)	428 (78.1)	700 (78.5)
	Outdoors	83 (39.7)	57 (41.6)	166 (28.2)	150 (26.0)	73 (13.3)	155 (17.4)
	Not indicated	13 (6.2)	2 (1.5)	43 (7.3)	22 (3.8)	47 (8.6)	37 (4.1)
Cause of injury, n (%)							
	In bed	1 (0.5)	1 (0.7)	5 (0.8)	2 (0.3)	9 (1.6)	6 (0.7)
	Simple fall	138 (66.0)	99 (72.3)	450 (76.4)	465 (80.6)	406 (74.1)	743 (83.3)
	Fall on stairs	11 (5.3)	3 (2.2)	23 (3.9)	27 (4.7)	20 (3.6)	31 (3.5)
	Traffic accident	34 (16.3)	28 (20.4)	45 (7.6)	44 (7.6)	24 (4.4)	38 (4.3)
	Could not be recalled	4 (1.9)	0 (0.0)	4 (0.7)	4 (0.7)	10 (1.8)	12 (1.3)
	Unknown*	21 (10.0)	6 (4.4)	62 (10.5)	35 (6.1)	79 (14.4)	62 (7.0)

*Not-indicated cases are included.

Note: Totals do not always sum to 100% due to rounding to the first decimal place.

**Figure 1:**
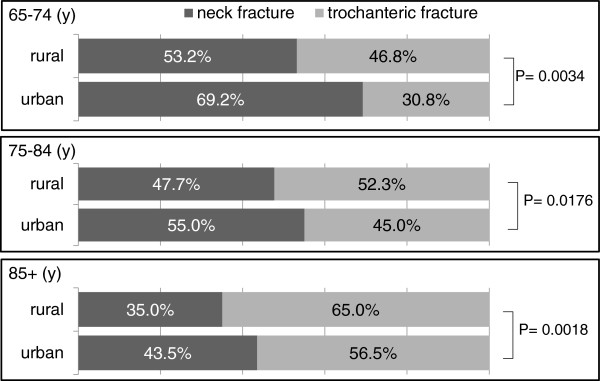
Comparison of neck-to-trochanteric fracture ratio between urban and rural areas in each age group.

In all age groups, the urban area showed a higher ratio of neck fracture than the rural area (Figure 1). Regarding the place of fracture, the percentage of fractures that occurred indoors (excluding not-indicated cases) increased with age: 57.8% (191/331) for subjects aged 65–74 years, 71.3% (785/1,101) for those aged 75–84 years, and 83.2% (1,128/1,356) for those aged ≥85 years (P = 0.0000 for all age groups). In terms of cause of injury, the percentage of simple falls (excluding unknown and not-indicated cases) also increased with age: 74.3% (237/319) for the age group 65–74 years, 85.6% (915/1,069) for the age group 75–84 years, and 88.5% (1,149/1,299) for the age group ≥85 years (P = 0.0385 for ages 75–84 years and ≥85 years, and P = 0.0000 for the other age groups).

The percentage of fractures sustained indoors between neck and trochanteric fractures was not significantly different (Table 2; P = 0.9820 for the age group 65–74 years, 0.2157 for the age group 75–84 years, and 0.0909 for the age group ≥85 years) or between urban and rural areas (Table 3; P = 0.1347 for the age group 65–74 years, 0.8388 for the age group 75–84 years, and 0.9418 for the age group ≥85 years) in any age group.

**Table 3:** Differences in place and cause of fracture between urban and rural areas in each age group

**Age group, y**			**65–74**		**74–84**		≥**85**
		**Urban**	**Rural**	**Urban**	**Rural**	**Urban**	**Rural**
No. of hip fractures		156	190	447	719	515	925
Place of injury, n (%)							
	Indoors	77 (49.4)	114 (60.0)	288 (64.4)	497 (69.1)	388 (75.3)	740 (80.0)
	Outdoors	68 (43.6)	72 (37.9)	118 (26.4)	198 (27.5)	79 (15.3)	149 (16.1)
	Not indicated	11 (7.1)	4 (2.1)	41 (9.2)	24 (3.3)	48 (9.3)	36 (3.9)
Cause of injury, n (%)							
	In bed	0 (0.0)	2 (1.1)	1 (0.2)	6 (0.8)	7 (1.4)	8 (0.9)
	Simple fall	101 (64.7)	136 (71.6)	348 (77.9)	567 (78.9)	399 (77.5)	750 (81.1)
	Fall on stairs	10 (6.4)	4 (2.1)	17 (3.8)	33 (4.6)	17 (3.3)	34 (3.7)
	Traffic accident	31 (19.9)	31 (16.3)	34 (7.6)	55 (7.6)	24 (4.7)	38 (4.1)
	Could not be recalled	2 (1.3)	2 (1.1)	3 (0.7)	5 (0.7)	10 (1.9)	12 (1.3)
	Unknown*	12 (7.7)	15 (7.9)	44 (9.8)	53 (7.4)	58 (11.3)	83 (9.0)

*Not-indicated cases are included.

Note: Totals do not always sum to 100% due to rounding to the first decimal place.

The percentage of fractures caused by simple falls (excluding unknown and not-indicated cases) was not significantly different between neck and trochanteric fractures (Table 2; P = 0.6629 for the age group 65–74 years, 0.8507 for the age group 75–84 years, and 0.1100 for the age group ≥85 years) or between the urban and rural areas (Table 3; P = 0.1234 for the age group 65–74 years, 0.5828 for the age group 75–84 years, and 0.3418 for the age group ≥85 years) in any age group.

**Figure 2:**
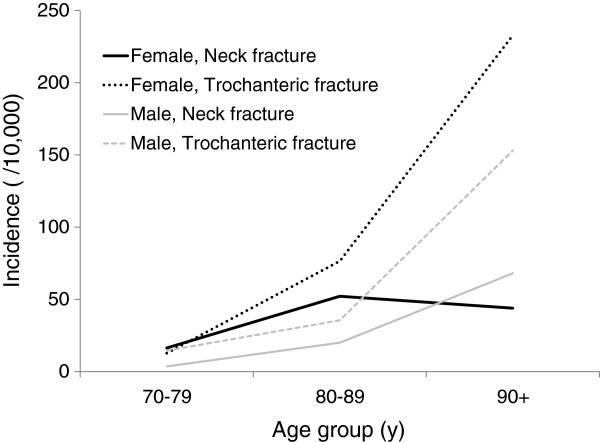
Age-related incidence of neck and trochanteric fractures in Tango medical district.

Men had almost the same or a slightly higher percentage of hip fracture incidence in Tango medical district (Table 4) than the results of the nationwide survey, with a contrasting low incidence in women in all age groups. In terms of fracture type, the neck fracture incidence among women aged ≥90 years was lower than that among women aged 80–89 years; it was also lower than that among men aged ≥90 years (Figure 2).

**Table 4:** Incidence of hip fracture in Tango medical district

	**Men**			**Women**		
**Age group, y**	**70–79**	**80–89**	≥**80**	**70–79**	**80–89**	**≥80**
Hip fractures/3 y	15	25	12	32	106	63
Population*	2,759	1,501	196	3,667	2,747	759
Incidence/10,000/y	18.12	55.52	204.08	29.09	128.63	276.68
Incidence in Japan/10,000/y [16]	18.12	61.03	146.62	39.71	157.14	313.58

*Source: National Census, 2010.

## Discussion

The percentage of women was approximately 80% for each fracture type in this study. The nationwide survey during the same period showed that the ratio of women was 80.3% (71,894/89,495) for neck fracture and 80.5% (84,329/104,712) for trochanteric fracture. The male-to-female ratio for each fracture type in Kyoto Prefecture is considered compatible with that in Japan as a whole.

The N/T ratio in Kyoto Prefecture was similar to that in Japan, which was approximately 0.84 [12]. These ratios did not significantly differ in any age between our results (Table 2) and those derived from the nationwide annual reports of the JOA: 1.53 (16,690/9,990) for the age group 65–74 years (P = 0.4114), 1.02 (39,036/38,524) for the age group 75–84 years (P = 0.9005), and 0.61 (34,069/56,198) for the age group ≥85 years (P = 0.8079). However, the difference in the ratio between regions was compatible with that obtained in the investigation of 2008 [15]. The N/T ratio was higher in the urban area and lower in the rural area compared with national statistics [12]. Our results showed that the urban area had a significantly higher N/T ratio than the rural area in all age groups.

The hip fracture incidence in 1 of the rural areas—Tango medical district—was lower than the national average in women. Considering the N/T ratio, this region was thought to have a lower hip fracture incidence, especially that of neck fracture. Although investigation of incidence was possible in only one part of the rural area, the tendency toward more neck fractures than trochanteric fractures was common among rural medical districts.

Most of the investigations in many countries, including the Nordic countries [17-20], France [21], the United States of America [22], Korea [23], the Canary Islands (tropical regions) [24], Switzerland [25], and Australia [26], have shown that urban areas have a higher incidence of hip fracture than rural areas, although 1 report pointed out the need for further investigation [27]. There are no reports showing a higher incidence of hip fractures in rural areas; therefore, hip fractures, especially of neck fractures, are believed to have a lower incidence in rural areas than in the urban areas in Kyoto Prefecture.

Fracture incidence in Kyoto Prefecture was previously shown to be slightly higher than the national average: men had a 1.00- to 1.09-fold increased incidence and women had a 1.10- to 1.19-fold increased incidence [16]. If the incidence is lower in the rural areas of Kyoto Prefecture, incidence in urban areas would be higher than the national average.

In terms of N/T ratio, the incidence of trochanteric fractures is more than that of neck fractures in individuals aged ≥75 years in Japan [10]. However, in the urban areas of Kyoto Prefecture, even in the age group of 75–84 years, the ratio of neck fractures was 55.0%. Therefore, occurrence of neck fractures in urban areas was expected to be higher than the national results obtained. Finsen showed that urban areas had a higher incidence of hip fracture than rural areas and that the number of neck fractures was quite remarkable [17].

In terms of lifestyle backgrounds, urban areas had a larger number of older individuals living alone, fewer individuals engaged in a primary industry such as agriculture, and more doctors per resident (Table 1). Lifestyle background may influence fracture occurrence through 2 major factors: external factors such as higher chance of falls, and internal factors such as bone fragility. For example, those living alone may have a higher chance of falling because of the need to go out to shop. In addition, differences in residential and traffic environments might influence external factors of injury. Rates of injuries caused by both indoor occurrence and simple fall were higher in the older age group, as has been reported previously [10]. However, there were no apparent differences in these factors between neck and trochanteric fractures in any age group. In addition, there were no differences in place or cause of injury between urban and rural areas in any age group. Based on this information, external factors did not appear to be a main cause for the difference in the N/T ratio between urban and rural areas.

With respect to internal factors, the mean number of hours of daylight per year reportedly has relevance in terms of fracture occurrence [28]. In Kyoto Prefecture, the mean total hours of annual sunshine increases inversely in proportion to latitude; in other words, the northern part receives approximately 1,500 h, while the southern part receives approximately 1,900 h (mean calculated from 1987–2010 according to climatological statistics information from the Japan Meteorological Agency). The N/T ratio had no relationship with regional differences in total hours of annual sunshine.

Medical intervention might influence occurrence of hip fracture. Interarea differences in the condition of community medicine are one of the most serious problems in Kyoto Prefecture. Our results indicate that the low N/T ratio in Tango medical district was because of the low incidence of neck fractures among women. Antiosteoporotic treatment will decrease fractures, but the number of doctors per capita in this district is the lowest in Kyoto Prefecture [15]. In contrast, other drugs besides corticosteroids have been reported to increase susceptibility to fracture [29,30]. Other medications are thought to induce a higher incidence of falling [31]. Thus, easy accessibility to medical care might increase neck fractures, resulting in a high N/T ratio in the urban area.

Many differences among the characteristics of each fracture type have been reported. Compared with neck fractures, trochanteric fractures have a strong relationship with bone density [32,33] and age [10,11,34,35], which is especially evident in women [4,5,21,36-38]. One report stated that women with trochanteric fracture had significantly lower serum 25(OH)D levels than those with neck fracture [14]. In neck fractures, factors other than bone density [33], such as greater height [39], high body fat percentage [40], high body mass index [41], and ongoing hypertensive treatment [41], are considered risk factors. In terms of height and weight, some reports have stated there are no differences between the 2 fracture types [41,42]. As an anatomic feature, patients with neck fracture reportedly have a longer hip axis length than those with trochanteric fracture [43]; however, other sources have reported no differences between the 2 fracture types [44,45]. In addition, different genetic factors have been shown to contribute to the occurrence of each fracture type [46].

Incidence of hip fracture in Okinawa [38] is reportedly higher than that in other Japanese prefectures [47,48]. The causes are believed to be the high obesity rate in youth and increased morbidity of lifestyle-related diseases in Okinawa [38]. According to a previous report [38], N/T ratio in Okinawa was higher than the national average [10] in all age groups.

The reason for the difference in N/T ratio between urban and rural areas in Kyoto Prefecture in this study remains unknown. However, past or present rural lifestyle factors such as agriculture as an occupation, may have some beneficial effects on bone fragility, especially in relation to neck fracture. Conversely, an urban lifestyle, which is strongly influenced by the Westernized lifestyle, may have a greater influence on the occurrence of neck fracture than on occurrence of trochanteric fracture. In fact, hip fractures have been estimated to increase with increasing N/T ratio over time in Japan [12].

It has been suggested that neck and trochanteric fractures should be addressed separately both clinically and epidemiologically [32,40]. When the major factors of each fracture become clear, more effective countermeasures can be devised through specific interventions such as lifestyle guidance and early medication for each population at risk.

Moreover, most investigations of fracture incidence have been conducted in rural areas, not in urban areas where a considerable number of patients must be treated in hospitals outside their place of residence. However, the present study showed a remarkable difference among regions even within the same prefecture. Therefore, results derived from surveys in rural areas might not be applicable even in adjacent urban areas.

This study has some limitations. First, fracture incidence was not investigated in major parts of the districts. Second, there were no data on medication or complications such as rheumatoid arthritis, which could have an influence on susceptibility to hip fracture. Third, it is not clear whether the study population is representative of Kyoto Prefecture. However, our results did show that the N/T ratio of Kyoto Prefecture was compatible with that derived from the nationwide annual reports of the JOA (2008–2010) in each age group.

## Conclusions

In conclusion, this study showed a difference in the incidence of neck and trochanteric fractures between urban and rural areas in Kyoto Prefecture. This difference may have been caused by the relatively low incidence of neck fracture in rural areas. Further surveys focusing on each type of fracture will be important to clarify trends and to develop a specific and effective strategy for the prevention of each fracture type.

## Competing interests

The authors declare that they have no competing interests.

## Authors’ contributions

MH, HF, NK, and TK contributed to the conception and design of the study. MH, TI, KU, KI, and TS participated in data collection. MH and NK performed the statistical analysis. MH, RT, and MN drafted the manuscript. All authors read and approved the final manuscript.

## Pre-publication history

The pre-publication history for this paper can be accessed here:

http://www.biomedcentral.com/1471-2474/14/304/prepub
